# Can-Seq: a PCR and DNA sequencing strategy for identifying new alleles of known and candidate genes

**DOI:** 10.1186/s13007-020-0555-0

**Published:** 2020-02-13

**Authors:** Jiangling Cao, Nial R. Gursanscky, Stephen J. Fletcher, Anne Sawyer, Mehershad Wadia, Lachlan McKeough, Marek Coleman, Uwe Dressel, Christelle Taochy, Neena Mitter, Hervé Vaucheret, Bernard J. Carroll

**Affiliations:** 1 School of Chemistry and Molecular Biosciences, The University of Queensland, Brisbane, QLD 4072 Australia; 2 Queensland Alliance for Agriculture and Food Innovation, The University of Queensland, Brisbane, QLD 4072 Australia; 3 Institut Jean-Pierre Bourgin, UMR 1318, INRA AgroParisTech CNRS, Université Paris-Saclay, 78000 Versailles, France

**Keywords:** Forward genetics, Candidate gene-Sequencing (Can-Seq), Post-transcriptional gene silencing (PTGS), Root-to-shoot transmission of PTGS (RTP), Map-based gene cloning, RNA interference (RNAi), Ethyl methanesulfonate (EMS)

## Abstract

**Background:**

Forward genetic screens are a powerful approach for identifying the genes contributing to a trait of interest. However, mutants arising in genes already known can obscure the identification of new genes contributing to the trait. Here, we describe a strategy called Candidate gene-Sequencing (Can-Seq) for rapidly identifying and filtering out mutants carrying new alleles of known and candidate genes.

**Results:**

We carried out a forward genetic screen and identified 40 independent *Arabidopsis* mutants with defects in systemic spreading of RNA interference (RNAi), or more specifically in *root-to-shoot transmission of post-transcriptional gene silencing *(*rtp*)*.* To classify the mutants as either representing a new allele of a known or candidate gene versus carrying a mutation in an undiscovered gene, bulk genomic DNA from up to 23 independent mutants was used as template to amplify a collection of 47 known or candidate genes. These amplified sequences were combined into Can-Seq libraries and deep sequenced. Subsequently, mutations in the known and candidate genes were identified using a custom Snakemake script (https://github.com/Carroll-Lab/can_seq), and PCR zygosity tests were then designed and used to identify the individual mutants carrying each mutation. Using this approach, we showed that 28 of the 40 *rtp* mutants carried homozygous nonsense, missense or splice site mutations in one or more of the 47 known or candidate genes. We conducted complementation tests to demonstrate that several of the candidate mutations were responsible for the *rtp* defect. Importantly, by exclusion, the Can-Seq pipeline also identified *rtp* mutants that did not carry a causative mutation in any of the 47 known and candidate genes, and these mutants represent an undiscovered gene(s) required for systemic RNAi.

**Conclusions:**

Can-Seq offers an accurate, cost-effective method for classifying new mutants into known versus unknown genes. It has several advantages over existing genetic and DNA sequencing approaches that are currently being used in forward genetic screens for gene discovery. Using Can-Seq in conjunction with map-based gene cloning is a cost-effective approach towards identifying the full complement of genes contributing to a trait of interest.

## Background

Forward genetics is a powerful tool for identifying genes and biochemical pathways that contribute to a biological trait [1, 2]. However, identification of the full repertoire of genes contributing to a trait is crucial to understanding the molecular basis of phenotypic variation [2–4].

Forward genetics begins with the identification of independent mutants showing phenotypic variation in a trait of interest [1]. Subsequently, complementation tests, which involve the crossing of all independent mutants with each other, followed by progeny analysis, can be used to identify groups of allelic mutants, i.e., mutants that carry an independent mutation in the same gene. For most traits, uncovering all of the genes involved requires the characterization of a large number of mutants, and the number of crosses required for the complementation tests can be prohibitive. The number of crosses needed to classify all mutants into allelic groups is equal to (n^2^ ‒ n)/2, where n is the number of independent mutants obtained from the forward genetic screen. For example, for a collection of 40 independent recessive mutants, 780 crosses and F1 progeny analyses would be required to classify all of the mutants into allelic groups. Furthermore, forward genetic screens are strongly biased towards the identification of large protein-coding genes, making it difficult to identify mutations in small genes that are contributing to the trait of interest. The recent discovery of large numbers of genes encoding microRNAs [5] or small peptides [6] emphasizes this bias against the identification of small genes in forward genetic screens.

New approaches are therefore needed to efficiently classify mutants into those having a mutation in a known or candidate gene versus a gene not yet known to contribute to the trait. With the advances in DNA sequencing technologies, whole genome or exome sequencing has become a complementary method to complementation tests for identifying new alleles of known and candidate genes [7–10]. However, the costs of these technologies remain prohibitive when analysing a very large number of independent mutants.

To overcome these technical limitations, and the problem of new alleles frequently arising in known genes and obscuring the identification of the full repertoire of genes contributing to a trait, we have developed a PCR and DNA sequencing approach called Candidate gene-Sequencing (Can-Seq). The approach allows the rapid classification of mutants into those carrying new alleles of known or candidate genes versus mutants carrying a novel mutation in an undiscovered gene. Can-Seq is a simple protocol based on deep sequencing of PCR-amplified known or candidate genes from bulks of independent mutants, followed by a bioinformatics pipeline to identify candidate mutations and PCR zygosity tests to identify the individual mutant carrying each candidate mutation.

In the pilot study described here, we used Can-Seq to identify homozygous candidate mutations in 28 of 40 independent *root-to-shoot transmission of post-transcriptional gene silencing *(*rtp*) mutants of *Arabidopsis*. Notably, these 28 independent *rtp* mutants with defects in systemic RNA interference (RNAi) carried homozygous nonsense, missense or splice site mutations in one or more of the 47 candidate genes analysed. The remaining 12 mutants did not carry a mutation in any of the 47 known or candidate genes, and these mutants represent unknown, yet to be discovered genes involved in systemic RNAi*.* Our results demonstrate that Can-Seq is an amenable, cost-effective and reliable approach for identifying mutants that carry new alleles in known and candidate genes, and by exclusion, for identifying mutants that carry a causative mutation in an undiscovered gene.

## Results

### Forward genetic screen for *rtp* mutants

To enable a forward genetic screen for mutants with defects in systemic RNAi, we developed a green fluorescent protein (GFP) reporter line in *Arabidopsis* called 10027-3, which mimics the phenotype of root-to-shoot, graft-transmissible post-transcriptional gene silencing (PTGS) [11]. In this reporter line, PTGS of *GFP* is initiated by the expression of a *GFP*-specific inverted repeat in the root tip as it forms during embryogenesis [11]. *GFP* silencing then spreads systemically up through the root and hypocotyl, and into the shoot apex such that all true leaves that form post-embryonically in 10027-3 wild-type plants show complete silencing of *GFP* [11].

**Fig. 1:**
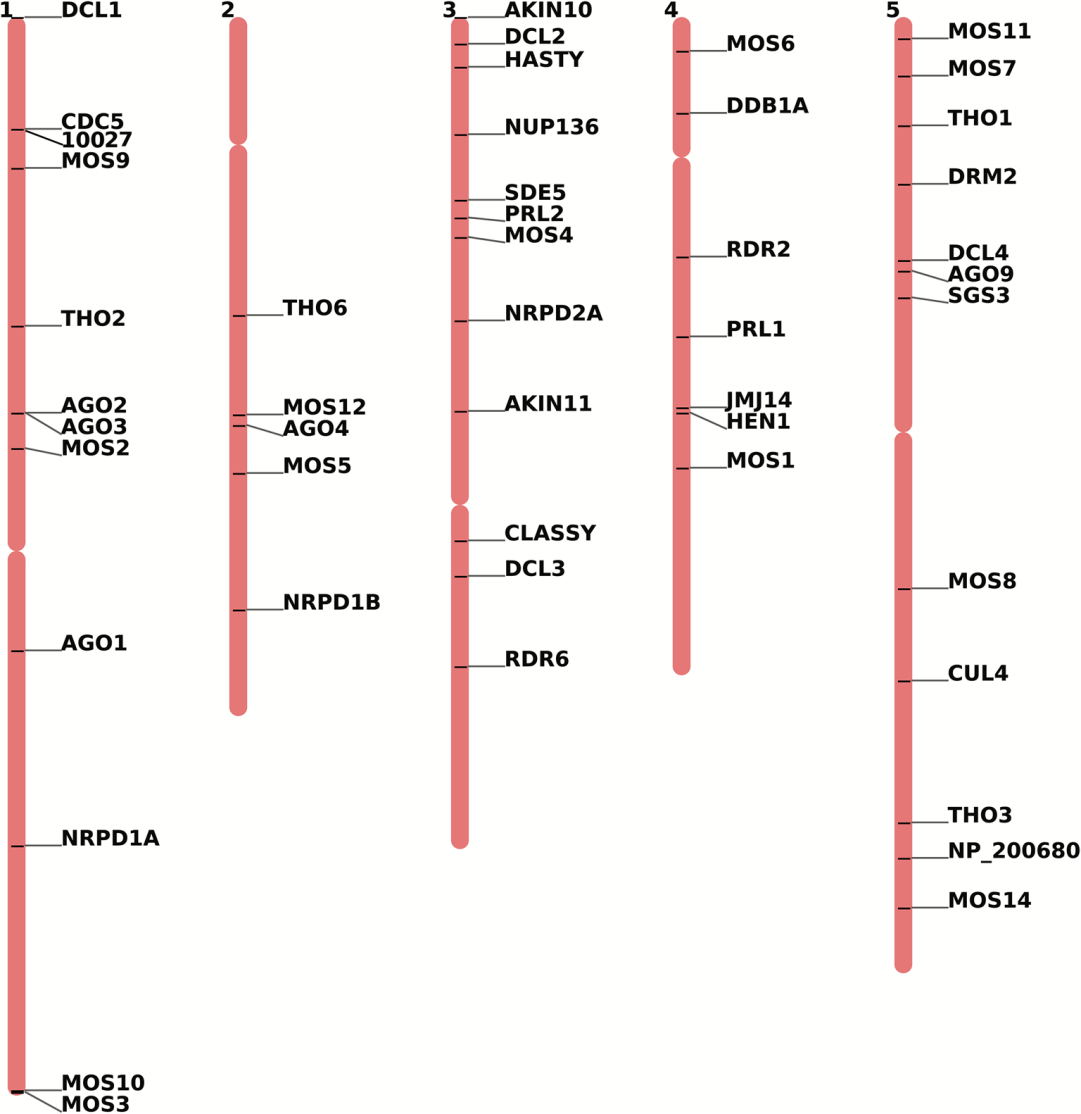
Chromosomal locations of the 47 candidate genes known or suspected to be involved in systemic RNAi in *Arabidopsis*. The position of the 10027-3 *GFP* reporter locus is indicated on the top end of chromosome 1 between *CDC5* and *MOS9* (10027)

Following ethyl methanesulfonate (EMS) mutagenesis of 10027-3 wild-type seeds, we identified 40 independent *root-to-shoot transmission of PTGS* (*rtp*) mutants with defects in systemic spreading of RNAi [11]. A large number of genes have been previously reported to play a role in RNAi in plants (e.g. [11–15].), and it was therefore expected that a large proportion of the *rtp* mutants would be caused by new mutations in these known or related candidate genes. Based on these earlier reports, we identified a suite of 47 genes that were known or suspected to be involved in systemic RNAi (Fig. 1; Additional file 1: Table S1).

### Identification of candidate mutations in independent *rtp* mutants

**Fig. 2:**
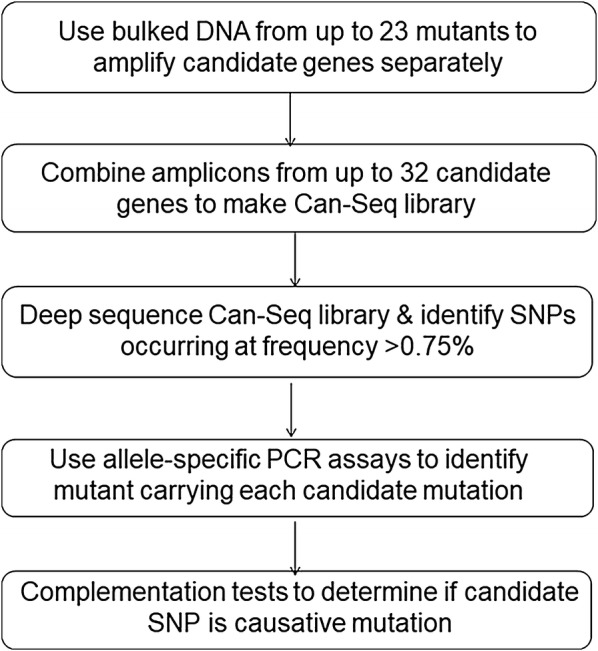
The Can-Seq workflow. Bulk DNA is prepared from leaf tissue of up to 23 independent mutants. Candidate gene PCR amplicons generated from this template are then combined in equimolar ratios and deep sequenced. Bioinformatic analysis using the Can-Seq script (https://github.com/Carroll-Lab/can_seq) allows for identification of C to T and G to A substitutions at frequencies above an arbitrarily set threshold of 0.75%; the expected frequency for a homozygous candidate mutation in a bulk of 23 independent mutants is 1 in 23 or ~ 4%. The individual mutant containing the candidate mutation is identified via allele-specific PCR assays. Complementation tests involving crosses between independent mutants carrying candidate mutations in the same gene can be used to resolve whether the EMS-induced nucleotide variant detected by Can-Seq is the causative mutation

The overall Can-Seq strategy and workflow is shown in Fig. 2. Equal amounts of leaf tissue from up to 23 independent *rtp* mutants were combined and used for bulk DNA extraction (Additional file 2: Table S2). Alternatively, equal amounts of purified genomic DNA from up to 23 independent *rtp* mutants were combined into a bulk DNA sample (Additional file 2: Table S2). This bulk DNA sample was then used as a template to separately amplify the full genomic locus of each of the 47 candidate genes. PCR amplicons from up to 32 candidate genes were then combined into a single sample for Illumina HiSeq 2000 library preparation, paired-end sequencing, and bioinformatics analysis. Thus, each Can-Seq library represented the amplified sequences of up to 32 candidate genes amplified from up to 23 independent *rtp* mutants.

Given that EMS mutagenesis generally results in G to A and C to T nucleotide transitions, we developed the bioinformatics component of the Can-Seq workflow to identify G to A and C to T substitutions that could result in nonsense, missense or splice site mutations in candidate genes (https://github.com/Carroll-Lab/can_seq). When a homozygous candidate mutation is present in one of the 23 mutants in a bulk, we would expect to detect this single nucleotide substitution at a frequency of 1 in 23, or ~ 4%, of the reads covering that locus. Similarly, we would expect a heterozygous mutation in a bulk of 23 mutants to be detected at a frequency of ~ 1 in 46, or ~ 2%, of the reads covering the variant nucleotide.

As internal positive controls, DNA from mutants with previously identified homozygous nucleotide substitutions in *RNA-DEPENDENT RNA POLYMERASE 6 *(*RDR6*), a gene known to be required for RNAi in *Arabidopsis* [12, 13], was included in each Can-Seq library*.* Indeed, each library always included two or three known *rdr6* alleles, namely *rtp2-1* (R376*; EMS#11), *rtp2-2* (W685*; EMS#19) and/or *rtp2-5* (W227*; EMS#153) ([11]; Table 1). The results of four representative Can-Seq libraries are shown in Additional file 2: Table S2, wherein these *rdr6* alleles were detected at a frequency that ranged from ~ 3 to 6% of reads, which approximated the expected frequency of ~ 4% (Additional file 2: Table S2). However, to ensure full recovery of all candidate mutations, including heterozygous mutations that were expected to occur at a frequency of ~ 2%, we arbitrarily set the threshold for recovery of candidate mutations at a frequency of 0.75% of coverage.

**Table 1:** Putative and confirmed *root-to-shoot transmission of PTGS *(*rtp*) mutations identified by Can-Seq and complementation tests

Candidate gene	Homozygous candidate mutation	*rtp* mutant	Putative *rtp* mutation	Causative *rtp* mutation	Mutant crossed to
*AGO1*	P204S	EMS#193	Yes	n.d.	EMS#152
	G277E	EMS#97	Yes	n.d.	EMS#152
	D769N	EMS#101	Yes	n.d.	EMS#152
	Intron 5 donor splice variant	EMS#152	Yes	Yes	EMS#193, EMS#97, EMS#101
*AGO9*	M261I	EMS#155	Yes	n.d.	EMS#140
	G853R	EMS#140	Yes	n.d.	EMS#155
*DCL2*^a^	W796*^a^	EMS#193^a^	Yes	Yes	EMS#149, *dcl2 (*Kas-1)
	A1098V^a^	EMS#149^a^	Yes	Yes	EMS#193, *dcl2 *(Kas-1)
*HASTY*	R544H	EMS#193	Yes	n.d.	EMS#155
	G1083S	EMS#155	Yes	n.d.	EMS#193
*JMJ14*	Q183*	EMS#38	Yes	n.d.	EMS#90
	G331E	EMS#148	Yes	n.d.	EMS#90
	Intron 2 acceptor splice variant	EMS#90	Yes	Yes	EMS#38, EMS#148
*NRPD1A*	S222F	EMS#193	Yes	n.d.	EMS#144
	R1174*	EMS#144	Yes	n.d.	EMS#193
*NRPD1B*^a^	A513T^a^	EMS#149^a^	Inconclusive	n.d.	EMS#193
	R1174*^a^	EMS#193^a^	Inconclusive	n.d.	EMS#149
*RDR6*^b^	G19E^b^	EMS#157^b^	No	n.d.	EMS#153
	W227*	EMS#153 (*rtp2-5*)^c^	Yes	Yes	EMS#19, EMS#94, EMS#146, EMS#159, EMS#157, *sde1-1*
	W685*^c^	EMS#19 (*rtp2-2*)^c^	Yes	Yes	EMS#153, *sde1-1*
	W764*	EMS#94	Yes	n.d.	EMS#153
	P1073L^b^	EMS#146^b^	No	n.d.	EMS#153
	R828K^c^	EMS#159 (*rtp2-6*)^c^	Yes	Yes	EMS#153, *sde1-1*
	R376*^c^	EMS#11 (*rtp2-1*)^c^	Yes	n.d.	*sde1-1*

Multiple independent candidate mutations were identified in all of the genes listed in the table, and complementation tests were used to identify putative and confirmed *rtp* causative mutations. We classified a candidate mutation as putative when an *rtp* phenotype was observed in the F1 progeny of a cross between a mutant and one other independent mutant carrying a homozygous mutation in the same candidate gene. On the other hand, we classified a candidate mutation as causative when an *rtp* phenotype was observed in the F1 progeny of a cross between a mutant and at least two other independent *rtp* mutants carrying a homozygous mutation in the same candidate gene. Based on the complementation tests shown in the table, most of the candidate mutations identified by Can-Seq were classified as at least putative *rtp* mutations. Furthermore, all seven candidate mutations that were tested by crossing to at least two other independent *rtp* mutants carrying mutations in the same candidate gene were confirmed to be causative *rtp* mutations. The two *dcl2* mutants in the Table were crossed to each other and also to the naturally occurring *dcl2* mutant in ecotype Kas-1, and in all cases, the F1 phenotype was mutant [11]. At least three F1 progeny plants were characterized for each combination of crosses. n.d., not determined

^a^Both EMS#193 and EMS#149 carried causative mutations in *DCL2* [11] and additional mutations in *NRPD1B* (also see Additional file 4: Table S4)

^b^The causative mutations in EMS#157 and EMS#146 did not map to the *RDR6* missense mutations (also see Additional file 4: Table S4)

^c^*rdr6* alleles described in Taochy et al. [11]

The PCR strategy was to amplify each candidate gene as a single amplicon if feasible, including at least 200 bp upstream and downstream of the translation start and stop sites, respectively. The full list of oligonucleotide primers used to amplify the 47 known and candidate genes are shown in Additional file 1: Table S1. Candidate mutations were identified in bulks of 20–23 mutants at a frequency that ranged from ~ 1 to 8% of the sequencing coverage for each particular locus. Subsequently, allele-specific, codominant cleaved amplified polymorphic sequence (CAPS) or derived CAPS (dCAPS) assays [16–18] were designed and used to identify the *rtp* mutant carrying each candidate nonsense, missense or splice site mutation. Using this strategy, 63 homozygous or heterozygous mutations were detected in one of the 40 *rtp* mutants (Additional file 3: Table S3), and of these, 43 were homozygous nonsense, missense or splice site candidate mutations in the respective mutant (Additional file 3: Table S3). The zygosity of each candidate mutation was based on genotyping at least six individual plants of the respective *rtp* mutant line. Multiple independent, homozygous candidate mutations were detected in seven of the 40 *rtp* mutants (Additional file 4: Table S4). Each of the 20 heterozygous candidate mutations were also detected in one of the 40 mutants (Additional file 3: Table S3), but these mutations were not characterized any further. Importantly, no false positive candidate mutations were identified by Can-Seq as all 63 homozygous or heterozygous mutations could be detected in one of the 40 *rtp* mutants (Additional file 3: Table S3).

To minimize the chance of false positive variant nucleotides arising in Can-Seq libraries, we included user-configurable filters in the bioinformatics pipeline. A false positive is considered a variant nucleotide identified in the bioinformatics workflow that cannot be experimentally validated in one of the mutants contributing to the Can-Seq library. By default, at least 200 reads must cover a variant position, with at least 30 reads containing the variant nucleotide. To eliminate false positive variant nucleotides associated with strand-specific sequencing errors, at least five reads of the variant nucleotide must align in both the forward and reverse orientation. Using these default filters to identify exon- and splice site-located canonical nucleotide substitutions (i.e. G→A; C→T), the frequency of the most abundant variant nucleotide compared to the reference genome nucleotide was zero for almost all nucleotides, with the exception of validated mutant-associated variants. As examples, Additional file 5: Figure S1 shows the frequency of variant nucleotides at every nucleotide of *AGO1* and *THO6* in multiple, independent Can-Seq libraries*.* Of all 47 candidate genes and mutant combinations analysed in our Can-Seq libraries, only a single exon-located nucleotide variant at position 1866 in *THO6* passed the alignment filters but fell under the arbitrarily set threshold variant frequency of 0.75% (Additional file 5: Figure S1). It is possible that such an identification represents a genuine heterozygous silent mutation in *THO6* given its appearance at a similar low frequency in independent Can-Seq libraries (Additional file 5: Figure S1). In view of the zero variant rate following filtration for all but this one nucleotide in the full collection of 47 known and candidate genes, it is very unlikely that this non-zero variant in *THO6* is a homozygous candidate mutation in a mutant represented in the Can-Seq libraries, which would be expected to occur at a frequency of ~ 4%.

The reproducibility of the frequency of variant nucleotides between independent Can-Seq libraries representing the same *rtp* mutants and candidate genes is clearly demonstrated in Additional file 2: Table S2 and Additional file 5: Figure S1.

### Complementation tests between mutants carrying candidate mutations in the same candidate gene

To confirm several of the candidate mutations as responsible for the *rtp* phenotypes, we conducted complementation tests. This involved crossing selected mutants to two other mutants carrying independent mutations in the same candidate gene, followed by F1 progeny analysis (Table 1). Based on backcrosses to the 10027-3 parent line, and BC1F1 and BC1F2 progeny analysis, we confirmed that the *rtp* phenotype was recessive for all mutants used in these complementation tests. Given a recessive nature of inheritance, crosses between allelic mutants produced F1 progeny with the *rtp* phenotype (i.e., defective in systemic PTGS), whereas crosses between non-allelic recessive mutants produced F1 progeny with a silencing phenotype similar to wild type.

**Fig. 3:**
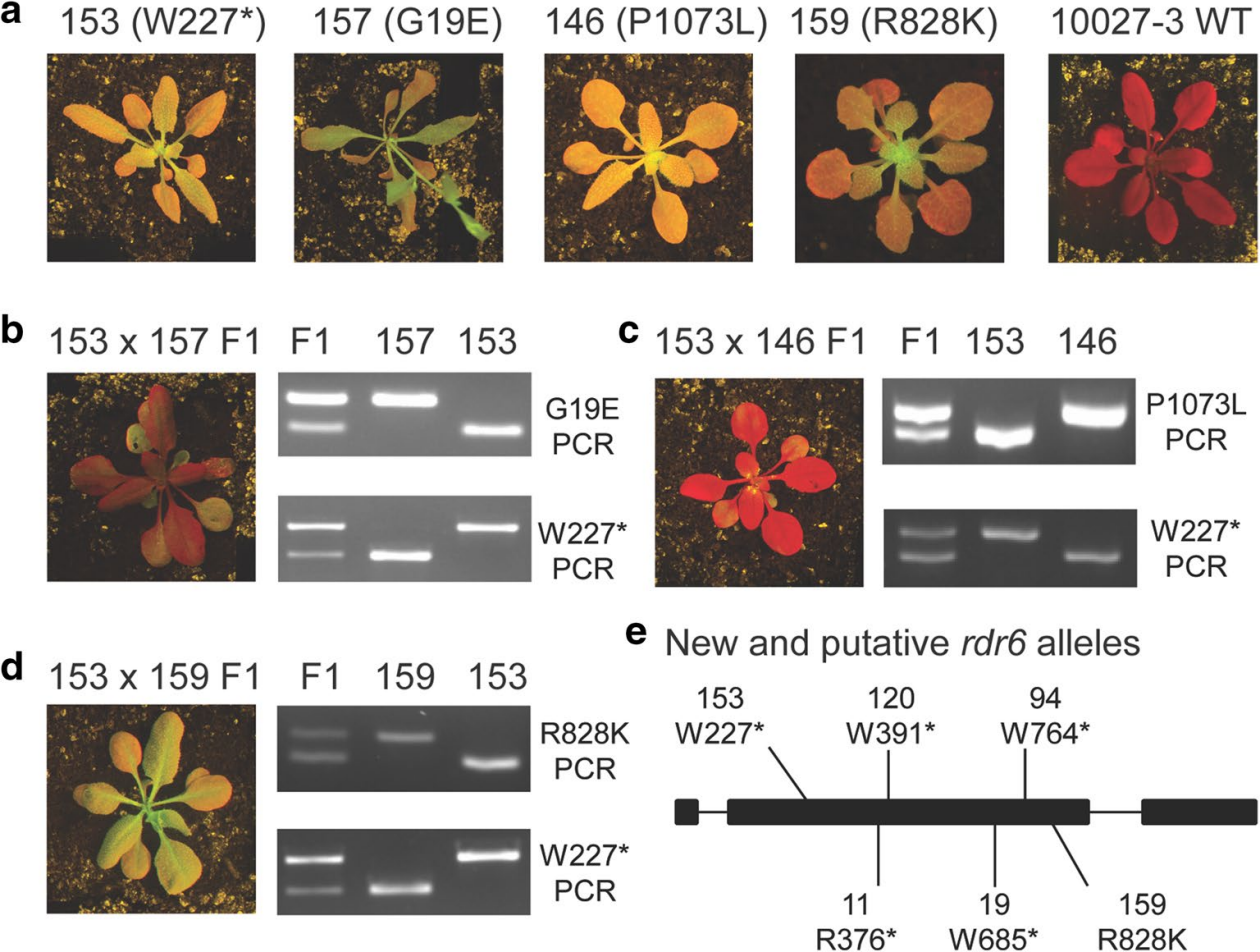
Missense *RDR6* mutation in EMS#159 (R828K), but not in EMS#146 (P1073L) or EMS#157 (G19E), is a putative new *rdr6* allele. **a** Rosette phenotypes of EMS#153 (W227*), EMS#157 (G19E), EMS#146 (P1073L), EMS#159 (R828K) and 10027-3 wild type (WT). The 10027-3 wild type shows systemic post-transcriptional gene silencing (PTGS) of *GFP*. Based on backcrosses to the 10027-3 wild type and analysis of the BC1F1 phenotype and BC1F2 segregation, the *rtp* phenotypes of EMS#153, EMS#157, EMS#146 and EMS#159 are inherited as recessive traits. **b**–**c** EMS#153 was complemented by EMS#157 and EMS#146, and F1 plants from these crosses showed almost complete systemic PTGS of *GFP*. **d** EMS#153 was not complemented by EMS#159 and F1 plants from this cross showed defective systemic RNAi of *GFP*. **e** Location of the new and putative *rdr6* alleles recovered by Can-Seq in the *RDR6* locus (AT3G49500). Exon and intron sequences are indicated by thick and narrow lines, respectively. Rosette images are of plants grown in soil under long days for four weeks after planting

Can-Seq identified five nonsense and three candidate missense mutations in *RDR6* (Additional file 3: Tables S3 and Additional file 4: Table S4). As expected, complementation tests based on crosses between several of the *rdr6* nonsense mutants and one of the first reported *rdr6* alleles, *sde1-1* [12], were consistent with the *rdr6* nonsense mutations being a causative mutation in each respective mutant (Additional file 6: Figure S2; Table 1). To determine if any of the three missense mutations in *RDR6* represented causative *rdr6* mutations, we crossed the missense mutants (EMS#157, EMS#146 and EMS#159) to the *rdr6* nonsense mutant EMS#153 (W227*), and analysed the F1 progeny for *GFP* silencing (Fig. 3). The EMS#153 × EMS#159 F1 plants were totally defective in *GFP* silencing, suggesting that the R828K missense mutation in *RDR6* is a causative mutation in EMS#159. However, to confirm this unequivocally*,* EMS#159 should be crossed to another *rdr6* nonsense mutant and the F1 phenotype confirmed to be mutant (Table 1). By contrast, the F1 plants from crosses between EMS#153 (W227*) and EMS#157 (G19E), or between EMS#153 (W227*) and EMS#146 (P1073L) showed almost complete silencing of *GFP* (Fig. 3). To confirm that the *RDR6* missense mutations in EMS#157 and EMS#146 were not the major causative *rtp* mutations in these mutants, we backcrossed the mutants to wild type and generated BC1F2 mapping populations. In both cases, the *RDR6* missense mutations did not co-segregate with the causative mutation in the EMS#157 or EMS#146 BC1F2 mapping populations (Additional file 7: Table S5), confirming that the G19E and P1073L missense mutations in *RDR6* were indeed silent mutations and not the causative *rtp* mutation in these mutants. We confirmed, however, that several of the candidate mutations in *RDR6* or other candidate genes were genetically linked to a defect in systemic RNAi (Additional file 7: Table S5).

## Discussion

### Optimisation of Can-Seq bioinformatics workflow

The Can-Seq bioinformatics workflow (https://github.com/Carroll-Lab/can_seq) was designed to be as simple and user-friendly as possible, and can be installed and operated on a consumer-grade PC. A single command instigates the workflow, which processes Can-Seq raw read files and GenBank-format reference files, carries out alignments, identifies variant nucleotides above the threshold frequency and the consequent amino acid changes, and outputs these data as both CSV and annotated GenBank files.

To date, we have produced Can-Seq libraries based on up to 23 mutants in a bulk DNA template for up to 32 candidate genes. In view of the potential risk of false positives, we feel we have approached the limit for the number of independent mutants that can be included in a single Can-Seq library. However, the number of candidate genes per Can-Seq library could be expanded further depending on the depth of sequencing. The bioinformatics workflow requires no modification should this be the case, and is independent of the number of candidate genes included and analysed in each Can-Seq library.

### Can-Seq identifies mutants carrying causative *rtp* mutations in novel genes

Can-Seq identified 63 candidate *rtp* mutations in homozygous or heterozygous configurations in one of 40 independent *rtp* mutants analysed (Additional file 3: Table S3). In total, 12 *rtp* mutants lacked a homozygous candidate mutation and 28 *rtp* mutants carried one or more homozygous candidate mutations (Additional file 4: Table S4). However, in the case of EMS#146, the homozygous candidate missense mutation in *RDR6* (P1073L) was not the causative mutation (Table 1; Fig. 3; Additional file 7: Table S5). Thus, 13 of the 40 *rtp* mutants either did not carry a homozygous candidate mutation, or carried a homozygous candidate mutation that was not linked to the causative mutation (i.e. EMS#146; Additional file 4: Table S4). Two of the *rtp* mutants carried candidate mutations in *DCL2*, which we subsequently showed by complementation tests to be the causative mutations in these mutants ([11]; Table 1). These were the first *dcl2* mutants to be recovered in a forward genetic screen, and the first demonstration that *DCL2* plays a crucial role in systemic RNAi in *Arabidopsis* [11]. Thus, Can-Seq can not only be used to recover new alleles of known genes for a trait of interest, but also to uncover a role for additional candidate genes not previously implicated in the trait.

**Fig. 4:**
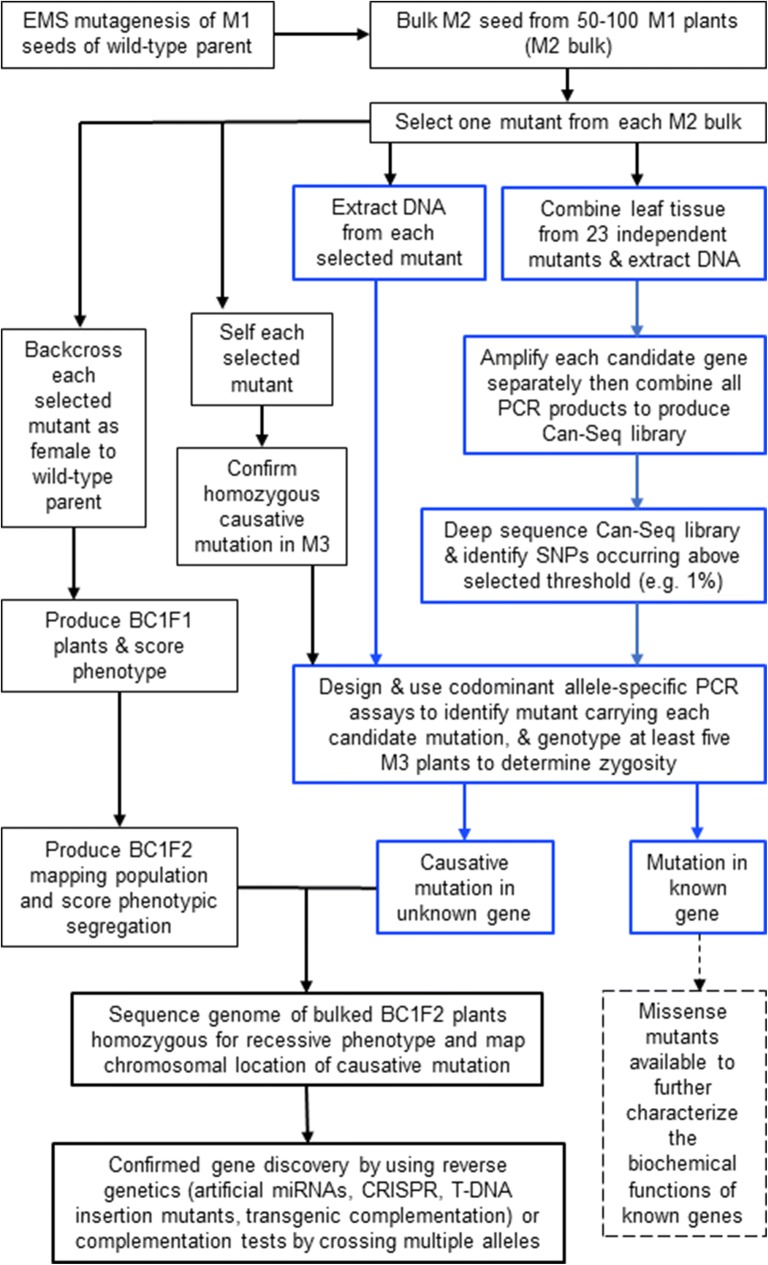
Pathway to gene discovery using Can-Seq in a forward genetic screen. The Can-Seq strategy shown in blue can be used in the M2 generation to identify mutants that carry a recessive candidate mutation in a gene known to contribute to the trait of interest. By exclusion, novel mutants carrying a causative mutation in an unknown gene can also be identified. For these novel mutants, BC1F2 mapping populations can be produced, and whole genome or exome sequencing of bulked BC1F2 mutant plants can be used to determine the chromosomal vicinity of the unknown gene contributing to the trait of interest. Reverse genetics on candidate genes in the chromosomal vicinity or complementation tests by crossing multiple alleles can then be used to reveal the identity of the new gene. Additionally, mutants identified by Can-Seq to carry new missense mutations in known genes can be confirmed by using complementation tests, and then potentially used to characterize the biochemical function of the protein encoded by the gene (dotted arrow and dotted box)

The 13 *rtp* mutants that either did not carry a homozygous candidate mutation, or carried a homozygous candidate mutation that was not linked to the causative mutation, therefore must harbour a causative mutation in a novel gene required for systemic PTGS (Additional file 4: Table S4). In future studies, these novel *rtp* mutations will be identified by using a map-based gene cloning approach, which would initially involve whole genome sequencing of bulk DNA from BC1F2 mutants to map the chromosomal vicinity of the causative mutation in each mutant ([19]; Fig. 4). Reverse genetics will then be used to identify the novel gene(s) involved in systemic PTGS (Fig. 4).

### Advantages of Can-Seq over exome capture sequencing

Currently, the main alternative to Can-Seq for identifying new alleles of candidate genes is whole genome or exome capture sequencing [8]. However, Can-Seq offers several advantages over exome capture sequencing, including (i) simpler and faster template preparation, (ii) lower costs per independent mutants (Additional file 8: Table S6), (iii) greater sequencing accuracy due to greater sequencing depth; Can-Seq delivered at least 60X coverage for each candidate gene compared to 20X coverage by exome sequencing [8], and (iv) less computationally intensive to mine and identify candidate mutations.

Particularly important for small budgets is the advantage of Can-Seq over whole genome or exome capture sequencing in cost per mutant (Additional file 8: Table S6). Excluding labour costs, the total cost of using Can-Seq to identify candidate *rtp* mutations in 40 mutants (including PCR, Illumina HiSeq 2000 sequencing and identifying the mutant carrying each candidate mutation) was conservatively estimated to about US$89 per mutant (Additional file 8: Table S6). The cost of whole genome or exome sequencing per mutant is conservatively estimated at ~ US$300 [20]. Thus, the cost of Can-Seq was about three times lower than the cost of whole genome sequencing or exome capture sequencing per mutant.

## Conclusions

New alleles arising in well-known genes obscure the discovery of the full repertoire of genes that are required for a trait. In particular, mutants of smaller genes are less likely to be recovered due to having fewer nucleotides. To address this limitation, future forward genetic screens could involve using Can-Seq to screen new mutants as soon as they are identified in order to filter out mutants that carry new alleles of known genes. This pathway to gene discovery using Can-Seq is illustrated in Fig. 4. Such a strategy would facilitate saturation mutagenesis and the discovery of the full repertoire of genes contributing to biological traits of interest.

## Methods

### Growth and EMS mutagenesis of *Arabidopsis*

*Arabidopsis* seedlings and plants were grown in long-day conditions (16 h light, 8 h dark) under fluorescent lighting (70–80 μmol/m^2^/s) at 21 °C. Seeds from the transgenic reporter line 10027-3 wild type in a Columbia (Col-0) genetic background were mutagenized with ethyl methanesulfonate (EMS) as previously described [21]. Approximately 30,000 M1 seeds were treated, then germinated on UC mix soil. M2 seeds were collected from batches of 100 M1 plants, then approximately 100 M2 seeds from each batch were sown on UC mix and screened for defects in systemic silencing of *GFP* at two weeks post-germination [11]. Only one mutant was selected per batch of 100 M1 plants, and so all 40 *rtp* mutants used in this study arose from independent mutation events.

### Photography and image analysis

Plant photographs were taken with an EOS 600D (Canon) digital camera with an orange filter for GFP visualisation. Blue light illumination was provided by six Dark Reader Hand Lamps (Clare Chemical Research). Images were uniformly adjusted for brightness and contrast with Adobe Bridge CS6.

### High-quality genomic DNA extraction

DNA extractions were performed on leaf tissue from individual mutants or on bulked leaves (each approximately 0.25 cm^2^) from up to 23 individual mutants. High-quality genomic DNA was extracted using the protocol of Carroll et al. [22], but with some modifications. Leaves were crushed using a plastic rod in a 2 mL Eppendorf tube containing liquid nitrogen, followed by the addition of 500 µL of pre-warmed nuclear lysis buffer (NLB; 0.2 M Tris–HCl pH 7.5, 0.05 M EDTA, 2 M NaCl, 2% (w/v) hexadecyltrimethylammonium bromide (CTAB), 0.6% (w/v) sodium sulfite) and 100 µL 5% (w/v) sarkosyl (*N*-lauryl-sarcosine). The tubes were then sealed and inverted gently 10 times. After incubation at 65 °C for 1 h, including inverting the tubes once every 10 min, 850 µL of phenol:chlorophorm:isoamyl alcohol (25:24:1) was added and mixed by inverting 60 times before centrifuging the tubes at full speed for 5 min. The aqueous phase containing the DNA (350 µL) was transferred to a clean 1.5 mL Eppendorf tube and the DNA was precipitated by the addition of 350 µL of isopropanol. DNA was pelleted by centrifugation at full speed for 5 min and washed twice in 500 µL of 70% ethanol. After removing all ethanol and air-drying in a laminar flow cabinet for 5 min, the DNA was resuspended in 50 µL of Tris–EDTA (10 mM Tris–HCL, 0.1 mM EDTA).

### PCR amplification for preparing PCR products for sequencing

For each candidate gene, PCRs were carried out using Phusion High Fidelity DNA polymerase (NEB). Each reaction contained 0.5 μM of each primer, 200 μM dNTPs, 3% DMSO, 0.4 U of Phusion polymerase, 1 × Phusion HF buffer, and approximately 100 ng of template DNA in a final volume of 20 μL. PCRs were run in heated-lid thermal cyclers, with optimal cycling conditions determined empirically. Typical cycling conditions were 35 cycles including a denaturing step of 98 °C for 15 s, an annealing temperature specific to each primer pair for 30 s, and an elongation step of 72 °C for 1 min per kb of amplicon. PCR products were electrophoresed on 0.7% agarose gels to confirm a single discrete product. PCR products were column purified using a QIAquick kit (Qiagen). PCR products for up to 32 candidate genes were bulked for DNA sequencing, ensuring equal DNA molarity of the PCR products from each candidate gene. Primers used to amplify the 47 known or candidate genes are listed in Additional file 1: Table S1.

### Deep sequencing

Library construction and deep sequencing were carried out by the Beijing Genomics Institute (BGI). Libraries were prepared for Illumina sequencing without the further use of PCR. Sequencing on the Illumina HiSeq 2000 platform generated 91 bp paired-end reads, which were provided in the FASTQ format. Approximately 15 million reads were generated for each Can-Seq library.

### Bioinformatics analysis

A workflow for identification of candidate gene mutations and annotation of GenBank format sequence files was developed as a custom Snakemake script (https://github.com/Carroll-Lab/can_seq). The process was implemented as follows: Trim-galore was used to remove adapter and low-quality portions of each read ([23]; https://www.bioinformatics.babraham.ac.uk/projects/trim_galore/). GenBank format reference files for each gene were converted to FASTA format using the SeqIO Biopython module (https://biopython.org/wiki/Biopython). Alignment of reads to reference sequences was carried out using Bowtie 2 [24], with variant positions identified using SAMtools mpileup output piped to VarScan [25, 26]. CSV files produced by VarScan were in turn parsed and used to generate a new annotated version of the original GenBank file for each gene, along with an additional simplified CSV output. Filters in the user-configurable config.json file were set to default values; for a nucleotide variant to be called, it must be a canonical EMS mutation (G→A or C→T), be present in at least 0.75% of reads covering the position, with at least 200 reads aligning to that position, of which at least 30 reads must contain the variant nucleotide and at least five reads of the variant nucleotide must align in both the forward and reverse orientation. Information provided in the output includes the location of the reference and variant nucleotide in the candidate gene, along with the abundance of the variant expressed as a percentage of total alignments at that nucleotide of the candidate gene.

### Mutation validation and zygosity assay

For the validation of each nucleotide variant, cleaved amplified polymorphic (CAPS) or derived cleaved amplified polymorphic (dCAPS) allele-specific PCR assays were carried out on genomic DNA extracted from individual mutant plants using *Taq* polymerase [16–18]. Each PCR reaction contained 0.5 μM of each primer, 200 μM dNTPs, 0.5 μM MgCl_2_, 0.25 U of *Taq* polymerase and approximately 100 ng of template DNA in a final volume of 10 μL in 1× PCR buffer (100 mM Tris pH 8.3, 500 mM HCl, 15 mM MgCl_2_ and 0.01% gelatin). Typical cycling conditions were 35 cycles including a denaturing step of 94 °C for 15 s, an annealing step of 60 °C for 15 s, and an elongation step of 72 °C for 3 min. After PCR amplification, PCR products were digested using the appropriate restriction enzyme for 2–3 h. Specific primers and restriction enzymes to detect each candidate mutation are listed in Additional file 3: Table S3. The zygosity of each candidate mutation was based on genotyping at least six individual plants of the respective *rtp* mutant line.

### Complementation tests

Complementation tests were performed by crossing independent *rtp* mutants containing homozygous candidate mutations in the same gene (see Table 1 for the crosses performed). Crosses were made using the protocol described by Weigel and Glazebrook [21]. The *GFP* silencing phenotype of F1 progeny was scored, and verification of the cross was carried out using the PCR zygosity assay described above. Each mutant used in complementation tests was also backcrossed to the 10027-3 wild type, and BC1F1 and/or BC1F2 progeny was scored for GFP expression and inheritance of the *rtp* phenotype. Genetic mapping of some candidate mutations was conducted on homozygous BC1F2 mutant plants using PCR zygosity tests described above.

## Supplementary information

**Additional file 1: Table S1.** List of genes known to be involved in post-transcriptional gene silencing (PTGS) in* Arabidopsis*, additional candidate genes and primers used to amplify them.

**Additional file 2: Table S2.** Observed and expected percent sequence reads of homozygous candidate mutations in Can-Seq libraries. Two or three* rdr6* nonsense mutants recovered in the forward genetic screen for mutants defective in systemic RNAi (11) were included in each Can-Seq library as a positive controls and are in bold.

**Additional file 3: Table S3.** CAPS/dCAPS PCR zygosity tests for detecting candidate mutations in* rtp* mutants. CAPS, cleaved amplified polymorphic sequences; dCAPS, derived cleaved amplified polymorphic sequences.

**Additional file 4: Table S4.** Homozygous candidate mutations in* root-to-shoot transmission of PTGS (rtp)* mutants identified by Can-Seq. The 40* rtp* mutants included in this study are listed in numerical order from left to right (EMS#11 to EMS#193). A blue background indicates mutants that do not carry a homozygous candidate mutation for the* rtp* phenotype, a red background indicates multiple homozygous candidate mutations were detected in a* rtp* mutant, a yellow background indicates a single homozygous candidate mutation was detected in a* rtp* mutant, and purple indicates a homozygous candidate mutation was detected in a* rtp* mutant but genetic mapping showed the candidate mutation was not closely linked to the causative mutation.

**Additional file 5: Figure S1.** The Can-Seq pipeline identifies exon- and splice site-located EMS-induced canonical nucleotide variants and no false positives. Following filtration, the frequency of the most abundant variant nucleotide is shown for every exonic and splice site nucleotide of* AGO1* and* THO6* in four and two Can-Seq libraries, respectively. *AGO1* and* THO6* were two of 32 candidate genes represented in Can-Seq libraries JC#4 and JC#5, and* AGO1* was one of 17 candidate genes in Can-Seq libraries JC#6 and JC#3. Furthermore, the same 23* rtp* mutants are represented in Can-Seq libraries JC#4, JC#5 and JC#6, and a different collection of 20* rtp* mutants are represented in Can-Seq library JC#3. For further details of these Can-Seq libraries see Additional file 2: Table S2. EMS-induced canonical variant nucleotides (i.e. G→A; C→T) are shown if (a) at least 200 reads cover the position, (b) at least 30 reads contain the variant nucleotide, (c) at least five reads containing the variant nucleotide align in both the forward and reverse orientation, and (d) the frequency of the variant nucleotide is greater than the arbitrary threshold of 0.75%, which is indicated by the horizontal red lines. The non-zero variant nucleotides in* AGO1* were subsequently detected as being homozygous (green) or heterozygous (blue) mutations in one of the* rtp* mutants that contributed to the Can-Seq library. Only the single nucleotide at position 1866 in the* THO6* alignment was identified as a non-zero variant that passed the Can-Seq pipeline filters except it fell under the 0.75% threshold, and the* rtp* mutant carrying this variant nucleotide was not determined (orange). This variant detected in* THO6* was the only non-zero variant nucleotide detected below the 0.75% threshold across all 47 candidate genes represented in our Can-Seq libraries, indicating that the Can-Seq pipeline identifies very few, if any, false positive variant nucleotides. Furthermore, this figure along with Additional file 2: Table S2, clearly demonstrates the reproducibility of the frequency of variant nucleotides between independent Can-Seq libraries representing the same* rtp* mutants and candidate genes. The Y axis shows the frequency of a variant nucleotide and the X axis shows the nucleotide position in* AGO1* or* THO6*.

**Additional file 6: Figure S2.** New nonsense alleles of* rdr6* in EMS#19 (W685*), EMS#94 (W764*) and EMS#153 (W227*).** A**. Rosette phenotypes of EMS#153 (W227*),* sde1-1* (10027-3* rdr6*), EMS#94 (W764*), EMS#19 (W685*), and 10027-3 wild type (WT). The 10027-3 wild type showed systemic post-transcriptional gene silencing (PTGS) of* GFP*. Based on backcrosses to the 10027-3 wild type and analysis of the BC1F1 phenotype and/or BC1F2 segregation, the* rtp* phenotypes of EMS#153, EMS#94 and EMS#19 are inherited as recessive traits.** B**. EMS#153 (W227*) was not complemented by* rdr6 (sde1-1)*, and F1 plants from this cross showed defective systemic PTGS. EMS#94 (W764*) (**C**) and EMS#19 (W685*) (**D**) were not complemented by EMS#153 (W227*), and F1 plants from each cross showed defective systemic PTGS of* GFP*.* RDR6* PCR genotyping assays are shown in the right panels of** B**,** C**, and** D**. The F1 phenotype and genotype was confirmed on at least three F1 individuals for each cross.** E**. Location of the new and putative* rdr6* alleles in the* RDR6* locus (AT3G49500). Exon and intron sequences are indicated by thick and narrow lines, respectively. Complementation tests for the new missense* RDR6/rdr6* alleles (EMS#157, EMS#146 and EMS#159) are shown in Figure 3. Rosette images are of plants grown in soil under long days for four weeks after planting.

**Additional file 7: Table S5.** Genetic mapping of Can-Seq candidate mutations relative to causative mutations in selected* root-to-shoot transmission of post-transcriptional gene silencing (rtp)* mutants.

**Additional file 8: Table S6.** Cost of using Can-Seq to screen 40* root-to-shoot transmission of post-transcriptional gene silencing (rtp)* mutants for candidate mutations in 47 genes.

## Data Availability

All data generated or analysis during this study are included in this published article and its additional information files. The Can-Seq workplan can be downloaded at https://github.com/Carroll-Lab/can_seq.

## Abbreviations

Can-Seq: Candidate gene-Sequencing
RNAi: RNA interference
*rtp*: *root-to-shoot transmission of post-transcriptional gene silencing*
EMS: ethyl methanesulfonate
PTGS: post-transcriptional gene silencing
GFP: green florescent protein
*RDR6*: *RNA-DEPENDENT RNA POLYMERASE 6*
CAPS: cleaved amplified polymorphic sequences
dCAPS: derived cleaved amplified polymorphic sequences
