# Supplementary material for: Can-Seq: a PCR and DNA sequencing strategy for identifying new alleles of known and candidate genes
Source: Plant Methods. 2020 Feb 13;16:16. doi: 10.1186/s13007-020-0555-0 (PMC7017465; doi:10.1186/s13007-020-0555-0)
Supplement: Supplementary file 1 — Additional file 1: Table S1. List of genes known to be involved in post-transcriptional gene silencing (PTGS) in Arabidopsis, additional candidate genes and primers used to amplify them. [file 13007_2020_555_MOESM1_ESM.docx]

**Table S1: List of genes known to be involved in post-transcriptional gene silencing (PTGS) in *Arabidopsis*, additional candidate genes and primers used to amplify them.**

| **Candidate gene** | **Locus** | **Primer name** | **Sequence** |
| --- | --- | --- | --- |
| *AGO1* | AT1G48410 | ago1 p1 F | CGTTTGTTCGGAGTTAGAGAGA |
|  |  | ago1 p1 R | CTGATTGTTTCCCCTCCAAA |
|  |  | ago1 p2 F | TTTTACAGACGAGAAAGGGAATTT |
|  |  | ago1 p2 R | AGCCTCAACAGAAGCCAGAG |
|  |  | ago1 p3 F | AGACGGTGCAACTCAATGAT |
|  |  | ago1 p3 R | TGGTTTATGCCCAGTTGATCT |
|  |  | ago1 p4 F | TTGGGTTTTAGCTTTAGCTTGC |
|  |  | ago1 p4 R | GAAAGGATCAAAGTCTGTTTTGAA |
| *AGO2* | AT1G31280 | ago2 p1 F | GCAGCAAAATTCTGAACACG |
|  |  | ago2 p1 R | CCTCTGCCTTTGTTGTGGAT |
|  |  | ago2 p2 F | TGTGACTTGGTTGAGGGACA |
|  |  | ago2 p2 R | TTCAGCTTGAGACCCAAAAC |
| *AGO3* | AT1G31290 | AGO3 Fwd | GGATCGAGGTGGTTACCGAGG |
|  |  | AGO3 Rvs | GAAGAGGATGAAGAGGCAACC |
| *AGO4* | AT2G27040 | ago4 p1 F | TGTCACCTTTCTGGGTTTGTC |
|  |  | ago4 p1 R | GCCAAGCAAAAGAGGTAAGG |
|  |  | ago4 p2 F | TGCTAGGGACCCTTATTCGAT |
|  |  | ago4 p2 R | GCAAAAACCATTTCCAAAAGA |
|  |  | ago4 p3 F | CGGCAACCTAATGATCAGTATCT |
|  |  | ago4 p3 R | CCTAAGCTGAAAGTCAGATAGCC |
| *AGO9* | AT5G21150 | AGO9 F P1 | gacgcgaatgattttgtttg |
|  |  | AGO9 F P2 | tcgacgtgtcctcaagaatc |
|  |  | AGO9 F P3 | ctgttttcccccttgaatgt |
|  |  | AGO9 R P1 | cttcggcttaccaacattga |
|  |  | AGO9 R P2 | accattcacaaggccctaaa |
|  |  | AGO9 R P3 | ttggttctccatcaaaagca |
| *AKIN10* | AT3G01090 | AKIN10 F P1 | atcgggtacgagagatccag |
|  |  | AKIN10 F P2 | agcaacataatgcgagatgg |
|  |  | AKIN10 R P1 | tacggggagtaccttcctga |
|  |  | AKIN10 R P2 | gtggcatgatgcactacaag |
| *AKIN11* | AT3G29160 | AKIN11 F P1 | gtttcagaccaaaaccacca |
|  |  | AKIN11 F P2 | taatgttatgcgggatggtc |
|  |  | AKIN11 R P1 | acatgtgcaggaatccagtg |
|  |  | AKIN11 R P2 | acccggcaaagtaaacactg |
| *CDC5* | AT1G09770 | CDC5seq1F | ggagcaatagctacttcaacaatg |
|  |  | CDC5seq1R | ttgccttggtgtttgtgagt |
|  |  | CDC5seq2F | aacagaaggcagtgctgcta |
|  |  | CDC5seq2R | tgcattgaaagagaggagga |
| *CLASSY* | AT3G42670 | Classy seq1 F | gttgctttcgcacattaacc |
|  |  | classy seq1 R | aacgcctcgttatccactct |
|  |  | Classy seq2 F | ttttcctgaaaacattcttgatg |
|  |  | Classy seq2 R | catcaaaggcatggtgaaac |
| *CUL4* | AT5G46210 | CUL4 F P1 | atttctcgggtcgggtcta |
|  |  | CUL4 F P2 | cagtctgacgttccggagta |
|  |  | CUL4 F P3 | ctcgaagggatgttcaaggt |
|  |  | CUL4 R P1 | acgttcgagaagttgccttt |
|  |  | CUL4 R P2 | acgtccaacgaggaaacaa |
|  |  | CUL4 R P3 | tgagaaccgaggaaaagtga |
| *DCL1* | AT1G01040 | DCL1 F P1 | ggatcgatagaccgtggaat |
|  |  | DCL1 F P2 | aacatgtgcctatgccttca |
|  |  | DCL1 F P3 | tgggtgcatttaccgatatg |
|  |  | DCL1 F P4 | gattcttgaagccttgactgc |
|  |  | DCL1 R P1 | ttccttgaacttgcttgtgc |
|  |  | DCL1 R P2 | catcttggtcagccttctca |
|  |  | DCL1 R P3 | agctcagctctctcgtagca |
|  |  | DCL1 R P4 | aagaccctttcttggcattg |
| *DCL2* | AT3G03300 | dcl2 p1 F | TGGTAGCAGTCAATAATCCCATAA |
|  |  | dcl2 p1 R | TTTTAACGGCACCTTTCACAT |
|  |  | dcl2 p2 F | CATGAACTCGAAACTCTAATGAA |
|  |  | dcl2 p2 R | GACCTTTCCACCATCATCAAA |
|  |  | dcl2 p3 F | TGCATCCAACATTTGCAGTT |
|  |  | dcl2 p3 R | AATTCCCTGTCATGCTCTGC |
|  |  | dcl2 p4 F | CAGAGCATGACAGGGAATTTG |
|  |  | dcl2 p4 R | TGAATGAATGCAATAAAGTTGACA |
| *DCL3* | AT3G43920 | dcl3 p1 F | TTTGACTAATTTTGTTGCAGTTTTT |
|  |  | dcl3 p1 R | TCAGAAAACTGTCTTTTCCCAAC |
|  |  | dcl3 p2 F | TTCCTATTATGTTTCAGAGTGCTTG |
|  |  | dcl3 p2 R | GAAACTGTTTATAACTAGGGATCAGAG |
|  |  | dcl3 p3 F | CAAGGCTTCTGTCTCACCTTG |
|  |  | dcl3 p3 R | AATGCAGGGGTTGAAAATGA |
|  |  | dcl3 p4 F | TCACACACTCCTCTCTTCGTG |
|  |  | dcl3 p4 R | GGAGTTGGTATACAAAAACCATTT |
| *DCL4* | AT5G20320 | dcl4 p1 F | GAAACACATGATTTCTGGTTCAG |
|  |  | dcl4 p1 R | CCCAAAACACAGCGTGATAG |
|  |  | dcl4 p2 F | GATATCTTTCCTAGAATTGAATACTGC |
|  |  | dcl4 p2 R | GCACAATGTGGTGAAAAGCA |
|  |  | dcl4 p3 F | TTCCAGCTCTTGATTCACCA |
|  |  | dcl4 p3 R | AGAGCCCGAAGGAGGAAATA |
|  |  | dcl4 p4 F | CCACTCTGTCTGCATGATGG |
|  |  | dcl4 p4 R | CAAGTAGTCCAGAACAGCATCC |
|  |  | dcl4 p5 F | TCCATCTATGGTAACCAAATTTCTC |
|  |  | dcl4 p5 R | TTTGATGCATGAGAGATGAGTAAAC |
| *DDB1A* | AT4G05420 | DDB1a Fwd | CTATCCTCCTGCTGCTTCTG |
|  |  | DDB1a Rvs | CAATCTACCCTCCCAATCCCAC |
| *DRM2* | AT5G14620 | DRM2 Fwd | CCAGTAAACTGACGACGATACAAGC |
|  |  | DRM2 Rvs | CGCAAAAAGCAAAAGAGAGTTAGGT |
| *HASTY* | AT3G05040 | hasty p1 F | ACCCAAAGCCAAATCAGAGA |
|  |  | hasty p1 R | TTTGCCAAGTCAAAGTGCTG |
|  |  | hasty p2 F | TGTTGCTGTGGTAATTTGCTG |
|  |  | hasty p2 R | CTGGCTGTCCATCACTGCTA |
|  |  | hasty p3 F | CGAGCACTTGTTTTCCTCTG |
|  |  | hasty p3 R | CCCTCATTTCTGGGGGTAAT |
|  |  | hasty p4 F | GCTACTCCGTGTTCTTCATTCC |
|  |  | hasty p4 R | CATGGAAGCTTTGGTTAGAGG |
| *HEN1* | AT4G20910 | hen1 p1 F | TCAGGGGTATGCACATTTTG |
|  |  | hen1 p1 R | CCAATGCAATATTCCTAAAATCC |
|  |  | hen1 p2 F | TCTCTTCTCTCCTACCGCTCA |
|  |  | hen1 p2 R | CATCCTCTGTTTACAGCCATGT |
|  |  | hen1 p3 F | GCTAGTTAGATGAATCTGTCGGTCT |
|  |  | hen1 p3 R | GCAAAAGCTCATGGCAAAAG |
| *JMJ14* | AT4G20400 | JMJ14 Fwd | CTTTGTTCTTCCTTAGGTCACGGGT |
|  |  | JMJ14 Rvs | GTGTTTATCTGCTTGCTCCTCCTTA |
| *MOS1* | AT4G24680 | MOS1_F | CTTTCTCCTCTTTCAACCAATCC |
|  |  | MOS1_R | CCACCAATTCCAAAATCCAAAC |
| *MOS2* | AT1G33520 | MOS2_F | GCCCATCTGCTTTACATTTATC |
|  |  | MOS2_R | CAGGCCCTTCTACACTATTTC |
| *MOS3* | AT1G80680 | MOS3_F | TAAGCAGAAGAGGAGGGAAAAC |
|  |  | MOS3_R | GGTCAGTAGCAATCAGAAACAAG |
| *MOS4* | AT3G18165 | MOS4 F P1 | gactatggcgacgaacaatg |
|  |  | MOS4 R P1 | ccacttgacaggagagcaga |
| *MOS5* | AT2G30110 | MOS5 Fwd | CCTTTCGTTCGCCCGTCTCTCT |
|  |  | MOS5 Rvs | CCAGGTTTGTTTGTTCAGAGTTTCA |
| *MOS6* | AT4G02150 | MOS6 F P1 | tgcgtggagaaaataaagga |
|  |  | MOS6 F P2 | acttccccaaacttcaggtg |
|  |  | MOS6 R P1 | accctaagagctggatgagg |
|  |  | MOS6 R P2 | aggagtgggcatgaagagat |
| *MOS7* | AT5G05680 | MOS7 Fwd | CAGATCAATACAACCCGTAGACC |
|  |  | MOS7 Rvs | CGCCGTGATTTTGCTACTTGA |
| *MOS8* | AT5G40280 | MOS8 Fwd | TGAATCTGGTTTGGTCTGGGC |
|  |  | MOS8 Rvs | GAAGCAGTTTCTGAGGCATTTG |
| *MOS9* | AT1G12530 | MOS9 Fwd | CAAGTGGATCGGTTCAAACAC |
|  |  | MOS9 Rvs | GATTTGGGGTTGGTAGAGGAAG |
| *MOS10* | AT1G80490 | MOS10 Fwd | CGACTCCAAATCACGAGAAGG |
|  |  | MOS10 Rvs | TTCAGCAATGTTGTCTGTGG |
| *MOS11* | AT5G02770 | MOS11 Fwd | TTCCAATCCTACATTCCTACTTCGC |
|  |  | MOS11 Rvs | CTCATGTACCCCTGTATGC |
| *MOS12* | AT2G26430 | MOS12 Fwd | TGTTGTCTCCCTGAATGTTGGC |
|  |  | MOS12 Rvs | ATGAAAATGTATCCGTTAGATGCG |
| *MOS14* | AT5G62600 | MOS14 Fwd | GTGTGGATCTGAAAGTGGAAATGG |
|  |  | MOS14 Rvs | CGGTTTAATGGCTCTCCTGG |
| *NP_200680* | AT5G58720 | NP_200680 F P1 | atttgctattcgcatgttgc |
|  |  | NP_200680 F P2 | gaacttttctggggtggaga |
|  |  | NP_200680 R P1 | taggattctggcgaatgttg |
|  |  | NP_200680 R P2 | acttatgccgttttgcattg |
| *NRPD1A* | AT1G63020 | nprd1b p2 F | TGCTGGCAAGAGAGGTTTTT |
|  |  | nrpd1a p1 F | GGTTAGTATTGGTCAGAGCGAGTT |
|  |  | nrpd1a p1 R | AACAGAAGTCGTTGGGAGGA |
|  |  | nrpd1a p2 F | GGATGGAGACACTGTGCTGA |
|  |  | nrpd1a p2 R | GGATTCTGCGCTCAGTTGTT |
|  |  | nrpd1a p3 F | ACCCATATAAAGCCACAAACA |
|  |  | nrpd1a p3 R | TACCGCAGAAGAGCCAGTCT |
| *NRPD1B* | AT2G40030 | nrpd1b p1 F | TACGCAGCTGAATCATCGAA |
|  |  | nrpd1b p1 R | TGAACAAAAAGAACGTCACCA |
|  |  | nrpd1b p2 R | CGAATGGAATATGACTTCAGCA |
|  |  | nrpd1b p3 F | CACCTATGGTGTCTCGCTTG |
|  |  | nrpd1b p3 R | ATACACTGCTCCCCCATCAA |
|  |  | nrpd1b p4 F | TTACCCAGTTCTGCTGGAAA |
|  |  | nrpd1b p4 R | ACGTCAGAAGTCGGCTCATT |
|  |  | nrpd1b p5 F | GATGCTCAAGAATCTTCCAAGT |
|  |  | nrpd1b p5 R | CGCCATTCTCTCCACTCTTT |
| *NRPD2A* | AT3G23780 | nrpd2a p1 F | TGCTTCGCTTAACCACTGAA |
|  |  | nrpd2a p1 R | TCCTGCTTTTTGACATCCAG |
|  |  | nrpd2a p2 F | TGTTTCTGTTTTGCTGTCCA |
|  |  | nrpd2a p2 R | ATCTCCAACCTTGCCGGTAT |
|  |  | nrpd2a p3 F | TTGTGGCTAATTTGGGTCGT |
|  |  | nrpd2a p3 R | CCAAACTGAACAAGCTCATCC |
|  |  | nrpd2a p4 F | TCTCGGGTACAACCAAGAGG |
|  |  | nrpd2a p4 R | GTCATCAGTGGCTCGGTTTT |
|  |  | NRPD2A seq 2R | cagaaacagtgatcggcatc |
|  |  | NRPD2A seq 2R | cagaaacagtgatcggcatc |
|  |  | NRPD2A seq2 F | agtggtgggtattcaaacga |
|  |  | NRPD2A seq2 F | agtggtgggtattcaaacga |
| *NUP136* | AT3G10650 | NUP136 F p1 | ttacgcagaaaggttcgttg |
|  |  | NUP136 F p2 | ttggtttcagattcgtctgtg |
|  |  | NUP136 F p3 | tgctgctgtatttcccaatatc |
|  |  | NUP136 R p1 | gggcccacagatcctatatc |
|  |  | NUP136 R p2 | ggtctgctgaagccatacct |
|  |  | NUP136 R p3 | aacaaaaagaccataaggctga |
| *PRL1* | AT4G15900 | prl1seq1F | tgtttaagaaaaggtaaaaccgtaa |
|  |  | prl1seq1R | tgctcgatatgcccagtaag |
|  |  | prl1seq2F | atgggatgtagcaactggag |
|  |  | prl1seq2R | caaaggtcccctctcaaaag |
| *PRL2* | AT3G16650 | PRL2 F P1 | tcttctcttccgtctttctcg |
|  |  | PRL2 F P2 | gattgcttctgtggctcttg |
|  |  | PRL2 R P1 | tccagtcgctacatcccata |
|  |  | PRL2 R P2 | ttttctggcttcgagtttga |
| *RDR2* | AT4G11130 | rdr2 p1 F | ACATGTTTGTGTTCTCCTACTCC |
|  |  | rdr2 p1 R | CATGAACAAGCGCATTGAGT |
|  |  | rdr2 p2 F | GACCTTTGCTTCTGCAGCTC |
|  |  | rdr2 p2 R | CAGGGTGAAGACATGGGTTT |
|  |  | rdr2 p3 F | TCGCAAGATTGATGAGGAAA |
|  |  | rdr2 p3 R | ACGCCAGCACTCCATTTATC |
| *RDR6* | AT3G49500 | rdr6seq1.2F | gatcggaaattgtcgtcattc |
|  |  | rdr6seq1.2R | catgaaagttacccgcaaaa |
|  |  | rdr6seq1F | agatcgcactgagattagagatg |
|  |  | rdr6seq1R | gagctcaacttctgggggta |
|  |  | rdr6seq2.2F | cgaccagtttttgatgcgta |
|  |  | rdr6seq2.2R | actgctcattcgccaagttt |
|  |  | rdr6seq2F | tgaagcttgttcaggaatgg |
|  |  | rdr6seq2R | tccatggctccatactcact |
|  |  | rdr6seq3F | tgctagtagcttctgccaatg |
|  |  | rdr6seq3R | tgggaggaagtgacacaaaa |
| *SDE5* | AT3G15390 | SDE5 F P1 | cactttgccattgatttgct |
|  |  | SDE5 F P2 | cttggcatatggctcttgtg |
|  |  | SDE5 R P2 | cacatatctttccgcacctg |
|  |  | SDE5 R P2 | agcaatcaggaggaagaggt |
| *SGS3* | AT5G23570 | SGS3 F P1 | cgaaggtttttgtgctatgg |
|  |  | SGS3 F P2 | aaagttctttggcagcttgg |
|  |  | SGS3 R P1 | acgatccgattatcctctgc |
|  |  | SGS3 R P2 | caccaacacgtcgctttaga |
| *THO1* | AT5G09860 | THO1 F P1 | atatccggttttggtgtcgt |
|  |  | THO1 F P2 | aacacctttgatgctcaacc |
|  |  | THO1 R P1 | tagcctctccctcttcttcg |
|  |  | THO1 R P2 | aagaaagggtcgacaaactga |
| *THO2* | AT1G24706 | THO2 F P1 | ttcgaaaagggaggagagtg |
|  |  | THO2 F P2 | tgcactgtgctctgatttacg |
|  |  | THO2 F P3 | agttttgttctggcctaccg |
|  |  | THO2 F P4 | gcagaatcctttgttgttgg |
|  |  | THO2 F P5 | ggcaggaagtgagactttacg |
|  |  | THO2 F P6 | catgtggcttctgtccgtag |
|  |  | THO2 F P7 | gacctgggttgtgagtgaga |
|  |  | THO2 R P1 | ttaatcgcacctgagaagca |
|  |  | THO2 R P2 | ccacaagagcagagaaacca |
|  |  | THO2 R P3 | agaagcaacgcaacagtttg |
|  |  | THO2 R P4 | tgcatatataaaacgcacacca |
|  |  | THO2 R P5 | tacaaacatcgcgcagtaca |
|  |  | THO2 R P6 | gcacctcgtttagaggaagc |
|  |  | THO2 R P7 | tcgttgaacaaaccaaagga |
| *THO3* | AT5G56130 | THO3 F P1 | ggagactgaggagcagaagg |
|  |  | THO3 F P2 | ttgagtcatatcgcggagac |
|  |  | THO3 R P2 | gtgtgggctgtgagagtgtc |
|  |  | THO3 R P2 | cgatcttaccattggcctct |
| *THO6* | AT2G19430 | THO6 F P1 | cctttgatactttcggtttgg |
|  |  | THO6 F P2 | tctgttgatcctcaggttgg |
|  |  | THO6 R P1 | ctgaacccgtcaatatctgg |
|  |  | THO6 R P2 | cagactaaaccggtccaatg |
