# Supplementary figures and images for: Can-Seq: a PCR and DNA sequencing strategy for identifying new alleles of known and candidate genes
Source: Plant Methods. 2020 Feb 13;16:16. doi: 10.1186/s13007-020-0555-0 (PMC7017465; doi:10.1186/s13007-020-0555-0)

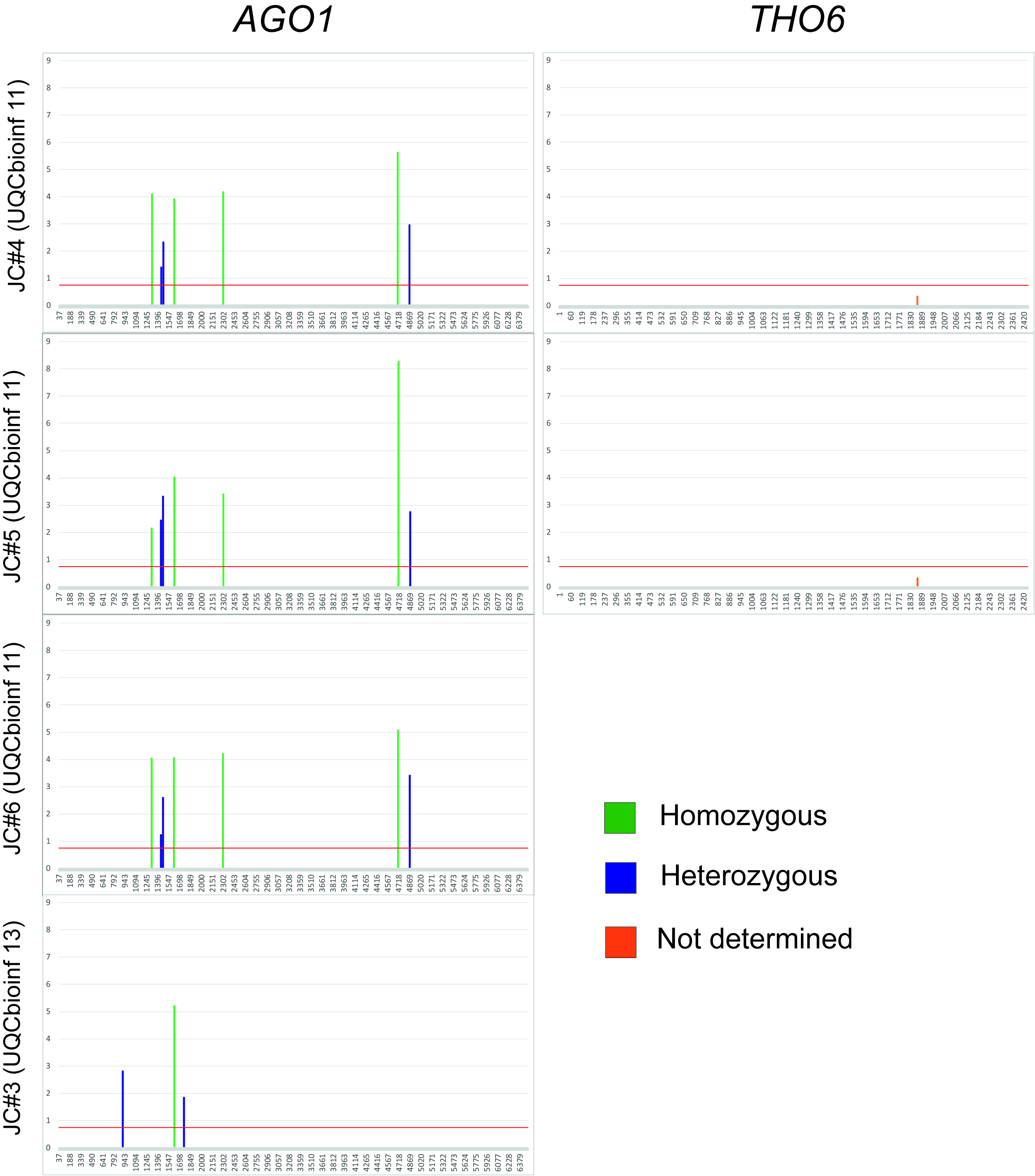

Supplement: Supplementary file 5 — Additional file 5: Figure S1. The Can-Seq pipeline identifies exon- and splice site-located EMS-induced canonical nucleotide variants and no false positives. Following filtration, the frequency of the most abundant variant nucleotide is shown for every exonic and splice site nucleotide of AGO1 and THO6 in four and two Can-Seq libraries, respectively. AGO1 and THO6 were two of 32 candidate genes represented in Can-Seq libraries JC#4 and JC#5, and AGO1 was one of 17 candidate genes in Can-Seq libraries JC#6 and JC#3. Furthermore, the same 23 rtp mutants are represented in Can-Seq libraries JC#4, JC#5 and JC#6, and a different collection of 20 rtp mutants are represented in Can-Seq library JC#3. For further details of these Can-Seq libraries see Additional file 2: Table S2. EMS-induced canonical variant nucleotides (i.e. G→A; C→T) are shown if (a) at least 200 reads cover the position, (b) at least 30 reads contain the variant nucleotide, (c) at least five reads containing the variant nucleotide align in both the forward and reverse orientation, and (d) the frequency of the variant nucleotide is greater than the arbitrary threshold of 0.75%, which is indicated by the horizontal red lines. The non-zero variant nucleotides in AGO1 were subsequently detected as being homozygous (green) or heterozygous (blue) mutations in one of the rtp mutants that contributed to the Can-Seq library. Only the single nucleotide at position 1866 in the THO6 alignment was identified as a non-zero variant that passed the Can-Seq pipeline filters except it fell under the 0.75% threshold, and the rtp mutant carrying this variant nucleotide was not determined (orange). This variant detected in THO6 was the only non-zero variant nucleotide detected below the 0.75% threshold across all 47 candidate genes represented in our Can-Seq libraries, indicating that the Can-Seq pipeline identifies very few, if any, false positive variant nucleotides. Furthermore, this figure along with Additional file 2: T [file 13007_2020_555_MOESM5_ESM.tif]

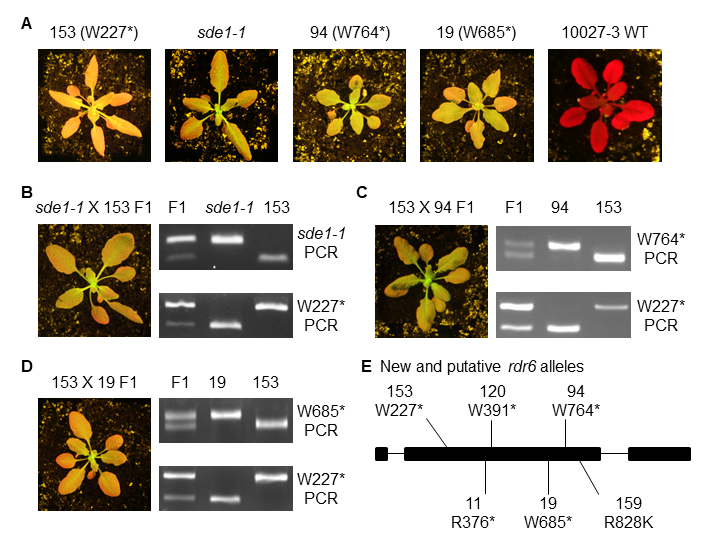

Supplement: Supplementary file 6 — Additional file 6: Figure S2. New nonsense alleles of rdr6 in EMS#19 (W685*), EMS#94 (W764*) and EMS#153 (W227*). A. Rosette phenotypes of EMS#153 (W227*), sde1-1 (10027-3 rdr6), EMS#94 (W764*), EMS#19 (W685*), and 10027-3 wild type (WT). The 10027-3 wild type showed systemic post-transcriptional gene silencing (PTGS) of GFP. Based on backcrosses to the 10027-3 wild type and analysis of the BC1F1 phenotype and/or BC1F2 segregation, the rtp phenotypes of EMS#153, EMS#94 and EMS#19 are inherited as recessive traits. B. EMS#153 (W227*) was not complemented by rdr6 (sde1-1), and F1 plants from this cross showed defective systemic PTGS. EMS#94 (W764*) (C) and EMS#19 (W685*) (D) were not complemented by EMS#153 (W227*), and F1 plants from each cross showed defective systemic PTGS of GFP. RDR6 PCR genotyping assays are shown in the right panels of B, C, and D. The F1 phenotype and genotype was confirmed on at least three F1 individuals for each cross. E. Location of the new and putative rdr6 alleles in the RDR6 locus (AT3G49500). Exon and intron sequences are indicated by thick and narrow lines, respectively. Complementation tests for the new missense RDR6/rdr6 alleles (EMS#157, EMS#146 and EMS#159) are shown in Figure 3. Rosette images are of plants grown in soil under long days for four weeks after planting. [file 13007_2020_555_MOESM6_ESM.tiff]
