# Supplementary material for: Can-Seq: a PCR and DNA sequencing strategy for identifying new alleles of known and candidate genes
Source: Plant Methods. 2020 Feb 13;16:16. doi: 10.1186/s13007-020-0555-0 (PMC7017465; doi:10.1186/s13007-020-0555-0)
Supplement: Supplementary file 7 — Additional file 7: Table S5. Genetic mapping of Can-Seq candidate mutations relative to causative mutations in selected root-to-shoot transmission of post-transcriptional gene silencing (rtp) mutants. [file 13007_2020_555_MOESM7_ESM.docx]

**Table S5: Genetic mapping of Can-Seq candidate mutations relative to causative mutations in selected *root-to-shoot transmission of post-transcriptional gene silencing (rtp)* mutants.** In all cases, the BC1F2 segregation ratio (1 mutant: 3 wild type) confirmed recessive inheritance of the mutant phenotype. The candidate mutation co-segregated with the causative mutation in EMS#94, EMS#38, EMS#90 and EMS#144, but not in EMS#157 or EMS#146.

| EMS mutant # | Candidate gene | Candidate *rtp* mutation | Observed segregation (mutant: wild type) | Expected segregation (1 mutant: 3 wild type) | Number of homozygous F2 mutants genotyped for candidate *rtp* mutation | Number of recombinants between candidate and causative mutations in F2 mutants | Genetic distance between candidate and causative *rtp* mutation (cM) |
| --- | --- | --- | --- | --- | --- | --- | --- |
| 94 | *RDR6* | W764* | 38: 92 | 32.5: 97.5 | 10 | 0 | 0 (<5 cM) |
| 157 | *RDR6* | G19E | 91: 255 | 86.5: 259.5 | 8 | 5 | 31.25 |
| 146 | *RDR6* | P1073L | 30: 96 | 31.5: 94.5 | 10 | 12 | 60 |
| 38 | *JMJ14* | Q183* | 56: 200 | 64: 192 | 40 | 0 | 0 (<1.25 cM) |
| 90 | *JMJ14* | intron 2 splicing defect | 64: 234 | 74.5: 223.5 | 49 | 0 | 0 (<1.02 cM) |
| 144 | *NRPD1A* | W541* | 62: 133 | 48.8: 146.2 | 49 | 0 | 0 (<1.02 cM) |
