# Supplementary material for: Can-Seq: a PCR and DNA sequencing strategy for identifying new alleles of known and candidate genes
Source: Plant Methods. 2020 Feb 13;16:16. doi: 10.1186/s13007-020-0555-0 (PMC7017465; doi:10.1186/s13007-020-0555-0)
Supplement: Supplementary file 8 — Additional file 8: Table S6. Cost of using Can-Seq to screen 40 root-to-shoot transmission of post-transcriptional gene silencing (rtp) mutants for candidate mutations in 47 genes. [file 13007_2020_555_MOESM8_ESM.docx]

**Table S6. Cost of using Can-Seq to screen 40 *root-to-shoot transmission of post-transcriptional gene silencing (rtp)* mutants for candidate mutations in 47 genes.**

| **Step** | | **Reagent / procedure** | **Description** | **Total cost for 40 mutants (US$)** | **Cost per mutant (US$)** |
| --- | --- | --- | --- | --- | --- |
| Sequence sample preparation | | Primer synthesis | 47 primer pairs, average 25 bases, 0.37 USD / base | 848 | 21.20 |
|  |  | Phusion high fidelity DNA polymerase | 47 PCR reactions, 0.37 USD / reaction | 16.8 | 0.42 |
|  |  | PCR product purification | 47 QIAquick columns, 2.20 USD /column | 100.8 | 2.52 |
| Illumina deep sequencing | | Library construction | 170 bp PCR-free | 1040 | 26 |
|  |  | Sequencing | Hiseq 91 PE |  |  |
| SNP validation | | CAPs / dCAPS markers | 53 primer pairs, average 25 bases, 0.37 USD / base | 956.4 | 23.91 |
|  |  |  | Restriction enzymes* | 600 | 15 |
|  |  |  | **Total** | **3562** | **89** |

* cost of restriction enzymes was conservatively estimated
